# Menopause education: An opportunity for cardiovascular prevention

**DOI:** 10.51866/lte.26-00040

**Published:** 2026-10-13

**Authors:** Anna Vittoria Mattioli, Giulia Renda, Sabina Gallina

**Affiliations:** 1 Department for Quality of Life Sciences, University of Bologna - Alma Mater Studiorum, Bologna, Italy.; 2 Department of Neuroscience, Imaging and Clinical Sciences, "G. D'Annunzio" University of Chieti and Pescara (Italy).

**Keywords:** Menopause, Cardiovascular disease, Health education, Hormone replacement therapy, Primary health care

**Dear Editor,**

We read with great interest the article by Puspitasari et al. entitled ‘Menopause matters: A community-based cross-sectional online survey among midlife women in Malaysia’.^1^ The authors provide valuable community-based evidence showing that menopause-related knowledge remains inadequate among midlife women. More than halfofthe respondents demonstrated poor knowledge, while a substantial proportion expressed negative attitudes towards menopause. Particularly relevant was the weak but significant positive correlation between knowledge and attitudes, suggesting that improved health literacy may contribute to a more informed and constructive experience of the menopausal transition.

We believe that these findings have implications extending beyond symptom awareness and psychological adaptation. Menopause education should increasingly be regarded as an opportunity for cardiovascular prevention.

Menopause is not only the end of reproductive life but also an important biological and cardiometabolic transition. The decline in ovarian hormone production is associated with changes in body fat distribution, insulin sensitivity, lipid profile, blood pressure regulation, endothelial function and vascular ageing.^2-4^ These changes may contribute to the progressive increase in cardiovascular risk observed during midlife and after menopause. Therefore, the menopausal transition represents a clinically relevant window in which cardiovascular risk factors can be identified early and preventive strategies can be initiated.

In this context, the finding that 53.3% of the respondents did not recognise the association between menopause and increased cardiovascular risk deserves particular attention.^1^ Awareness was also limited regarding the association of menopause with diabetes, weight gain and changes in body composition. This fragmented knowledge may reduce women’s ability to understand the long-term implications of menopause and may delay cardiovascular risk assessment, lifestyle modification and appropriate medical consultation.^5-7^

Menopause education could therefore extend beyond the description of hot flushes, sleep disturbances and menstrual cessation to include information on long-term cardiometabolic health. It may include clear information regarding blood pressure, glucose metabolism, lipid levels, body weight, smoking, physical activity, diet and family history of cardiovascular disease.^5-8^ Women should also be informed that menopause may represent an appropriate time to reassess their overall cardiovascular risk and discuss personalised preventive interventions with primary care professionals.

Menopausal hormone therapy is one component of this broader educational framework. In the study, 62.7% of the participants recognised its use for menopausal symptoms.^1^ Education should clarify that treatment requires an individualised benefit-risk assessment based on age, time since menopause, symptom burden and cardiovascular and thrombotic profiles. In appropriately selected symptomatic women, particularly those younger than 60 years or within 10 years of menopause onset, it remains the most effective treatment for vasomotor symptoms but should not be prescribed solely for the primary prevention of cardiovascular disease.^2,9^ Its potential influence on cardiometabolic measures, including serum uric acid levels, also supports documenting treatment exposure during cardiovascular assessment.^10^ The aim should be informed, shared decision-making rather than routine use or indiscriminate avoidance.

Primary care has a central role in this process. Family physicians are ideally positioned to transform the menopause consultation from a symptom-focused encounter to a broader preventive assessment. A structured midlife consultation could include vasomotor and genitourinary symptom evaluation, blood pressure measurement, weight and waist assessment, lipid and glucose screening, smoking and lifestyle counselling, mental health evaluation, bone health assessment and menopausal hormone therapy discussion when appropriate.

Educational interventions should also be culturally sensitive. The negative perceptions described in the study, including the association ofmenopause with loss ofyouth, femininity or productivity, may discourage women from seeking professional support.^1^ Normalising menopause as a physiological transition while acknowledging its clinical consequences may help reduce stigma and improve engagement with preventive health care.

Future research should evaluate whether structured menopause education leads not only to better knowledge and attitudes but also to measurable changes in clinical behaviour. Relevant outcomes should include assessment of cardiovascular risks, lifestyle modification, treatment-seeking, adherence to preventive recommendations and control of cardiometabolic risk factors.

The study by Puspitasari et al. highlights an important gap in women’s health literacy. Its findings could encourage a broader change in perspective: Menopause education is not merely a strategy to improve symptom recognition. It is an opportunity to identify cardiometabolic vulnerability, promote informed therapeutic choices and strengthen cardiovascular prevention throughout midlife.
